# Effect of a supportive-educational program, based on COPE model, on quality of life and caregiver burden of family caregivers of heart failure patients: a randomized clinical trial study

**DOI:** 10.1186/s12912-024-01709-2

**Published:** 2024-01-26

**Authors:** Atefeh Alaei, Sima Babaei, Sedigheh Farzi, Zahra hadian

**Affiliations:** 1 Master of Science of Medical and Surgical Nursing, Faculty of Nursing and Midwifery, Isfahan University of Medical Sciences, Isfahan, Iran; 2 Nursing and Midwifery Care Research Center, Faculty of Nursing and Midwifery, Isfahan University of Medical Sciences, Isfahan, Iran

**Keywords:** COPE model, Quality of life, Caregiver burden, Family caregivers, Heart failure

## Abstract

**Background:**

Heart failure (HF) is one of the most common and spreading diseases worldwide. As HF symptoms progress, it is affected the quality of life and the caregiver burden of the family. The present study aimed to determine the effectiveness of the supportive-educational program, based on the COPE (creativity, optimism, planning and expert advice) care model, on the quality of life and caregiver burden of family caregivers of HF patients.

**Method:**

This clinical trial was conducted on 90 caregivers of HF patients admitted to Isfahan in 2021. The samples were divided into experimental (*n* = 45 people) and control (*n* = 45 people) groups based on random allocation (card method). The experimental group received problem-solving skills based on the four components of the COPE model in six sessions during one month. to collect data, the 36-Item Short Form Survey (SF-36) and the Zarit Burden Interview (ZBI) were used in two groups before, after and three months after the intervention.

**Results:**

There was a significant difference between the experimental and control groups regarding gender, but This confounding factor had no significant effect on the two components of quality of life and caregiver burden. There was a significant difference between the two groups in terms of the mean score of quality of life immediately(75.99),and three months after the intervention (78.78) (*P* < 0.05) and the mean score of care burden, immediately (16.60) and three months after the intervention (12.73) (*P* < 0.05).

**Conclusions:**

One of the important duties of nurses is to implement educational-supportive programs for patients after the discharge of patients, These programs are family-oriented remotely for their caregivers.

**Trial registration:**

This study was registered by the Iranian Registry of Clinical Trials with decree code: IRCT20211128053202N1on 2022–02-20.

## Background

Heart failure (HF) is one of the most common cardiovascular disorders, a chronic, progressive and debilitating disorder whose prevalence increases with age [1, 2]. According to the American Heart Association, HF is a complex clinical syndrome caused by a functional or structural disorder of ventricular filling or cardiac output [3]. HF symptoms progress over time, which not only impose pressures and cause disability in the affected person, but also cause pressures on the resources available in the society and their families [4]. According to recent studies, HF affects about 64.3 million people worldwide and about 1.3 -6.7 million people in South Asia [5, 6], and these figures in developed countries range between 1 to 2% [5]. More than 40 million adults in the United States serve as family caregivers. In fact, family caregivers are partners, relatives, or friends who provide care over months to years that requires significant time and involves performing a wide range of physical, social, emotional, or financial tasks [7]. The duties of family caregivers of HF patients have been valued at 7.9 billion dollars due to providing multiple healthcare services in the United States [8].

Paying attention to the quality of life of caregivers of patients has an impacts on the recovery rate [9]. Therefore, quality of life and coping strategies seem essential in the caregivers of chronic patients [10]. Quality of life is considered a subjective evaluation of a person in relation to the world, according to the conditions she is currently experiencing [11]. Quality of life is a complex and multidimensional concept that does not have a single definition. However, the World Health Organization defines the quality of life as an individual's perception of their position in life in the context of the culture and value systems in which they live and about to their goals, expectations, standards and concerns. The term quality of life is a broad-ranging concept affected in a complex way by the person's physical health, psychological state, level of independence, social relationships with the characteristics of their environment [9]. The psychological responses of family caregivers to the crisis caused by a family member suffering from HF due to psycho-social limitations can lead to secondary social stressors and create tensions that increase physical, mental and financial burdens. These families may neglect their health and care, as a result of which their quality of life decreases [12].

The caregiving burden increases with caregiving hours; it is estimated that 40% of caregivers are in heavy caregiving situations [13]. Pressure or caregiver burden describes the physical, psychological and socioeconomic reactions imposed on the caregiver during the patient's care [14]. The increased caregiver burden in caregivers causes changes in their lifestyle and subsequent consequences such as inadequate patient care, patient abandonment, family isolation, weaker performance, decreased well-being, decreased self-control, more psycho-social stress, being frustrated from social support, disrupted family relationships, all of which reduce the quality of life of caregivers and even patients themselves [15, 16].

Therefore, these caregivers can be called vulnerable people or "invisible patients" who need the attention or interventions of medical staff and social workers [17]. Caregiver burden and coping strategies include providing information to the caregivers according to their needs and understanding [18], providing solutions to reduce the psycho-social pressures on the caregivers and supporting the caregivers through telephone [19]. One of these measures is family-centered interventions, which significantly impact families with chronic patients such as HF.

There are many supportive educational approaches to improve various aspects of patient caregivers, including quality of life and care burden, one of the most effective of which is the cognitive-behavioral problem-solving approach developed by Hoots et al. (1996) based on the stress and adaptation model and the family system theory, which is called COPE (Creativity, Optimism, Planning and Expert advise) [20]. The COPE model is a systematic approach to problem solving that enables chronically ill patients and their caregivers to interact with their healthcare providers, develop various skills for managing emotions and functioning, and learn useful interactions for physical comfort [21]. This supportive-educational approach improves the quality of patient care and improves the caregiver's physical and mental health. As symptoms of heart failure progress, self-care in these patients becomes poorer and they depend on informal caregivers, which in turn increases the importance of the caring-supporting role of the family and friends of these patients.

Since nurses are at the fore front of in contact with patients and their family members, they can provide the necessary knowledge, skills and support for these patients and their informal caregivers to maintain and improve the quality of care when the nurse is not present and in out-of-hospital environment [22]. Literature review showed no study on the effectiveness of the COPE model on the quality of life and caregiver burden of caregivers of HF patients in Iran. Therefore, this study was done study was to investigate the effectiveness of the supportive-educational program, based on the COPE care model, on the quality of life and caregiver burden of family caregivers of HF patients.

## Methods

The current research is a *single-blind study,* two-group, three-stage clinical trial that was approved by the Ethics Committee of Isfahan University of Medical Sciences (IR.MUI.NUREMA.REC.1400.196) on 04/10/2021 and registered with the code IRCT20211128053202N1in 20/02/2022 in Iranian Registry of Clinical Trials.

### Information about the participants

The study population included family caregivers of HF patients. Before random sampling, an interview was conducted with 30 caregivers of HF patients to obtain information about the content of the educational booklet based on their needs, limitations, deficiencies, disabilities and abilities. Inclusion criteria included willingness to participate in the study, the age over 18 years and under 65 years, being no member of the health team, being able to read and write in Persian language, not participating in a similar study simultaneously, not taking care of another chronically ill person at the same time, no self-reported psychiatric disorder, being the main caregiver, caring an HF patient who referred to the relevant center for treatment, and being able to use WhatsApp messenger. Exclusion criteria also included unwillingness to participate in the study, any problems in such a way that the caregiver was unable to continue participating in the study, the patient died during the study, not participating in one telephone session or one or multiple hospital visit sessions, not completing more than 5% of the items in the questionnaires. The sample size was estimated to be 90 people, taking into account a 10% dropout.

In this research, the sample size was calculated using the following formula [23]:

$${{\text{m}}}_{{\text{VariancesUnequal}}} = \left(\frac{\uptau +\mathrm{ \varphi }}{\mathrm{\varphi }}\right) \frac{{\left({{\text{z}}}_{1 -\mathrm{ \alpha }/2} + {{\text{z}}}_{1 -\upbeta }\right)}^{2}}{{\Delta }_{{\text{VariancesUnequal}}}^{2}} + \frac{\left({\uptau }^{2} + {\mathrm{\varphi }}^{3}\right) {{\text{z}}}_{1 -\mathrm{ \alpha }/2}^{2}}{{2\mathrm{\varphi }\left(\uptau +\mathrm{ \varphi }\right)}^{2}}.$$

That:

$${\Delta }_{{\text{VariancesUnequal}}} = \frac{{\mu }_{2} - {\mu }_{1}}{{\sigma }_{1}}$$

$${\sigma }_{2}^{2} = {\tau \sigma }_{1}^{2}$$

Ingredients of the Formula:

1. α is the confidence coefficient, is considered equal to 0.05.
2. β-1 is the statistical power that is considered equal to 0.80.
3. Note that z is the normal test's probability value,, which is calculated from the normal probability table.
4. iµ is the average of the investigated parameter in the two groups.
5. 1σ is the standard deviation in the experimental group.
6. φ is the sample volume ratio in the control group to the experimental group, which is considered equal to one here. That is, the two groups have the same sample size.
7. τ is the variance ratio of the control group to the experimental group.
8. ∆ is the effect size. According to the article by DeHol et al. [24], the sample size in each group (experimental group and control group) was calculated as 41 people.

### Processes and interventions

**Fig. 1:**
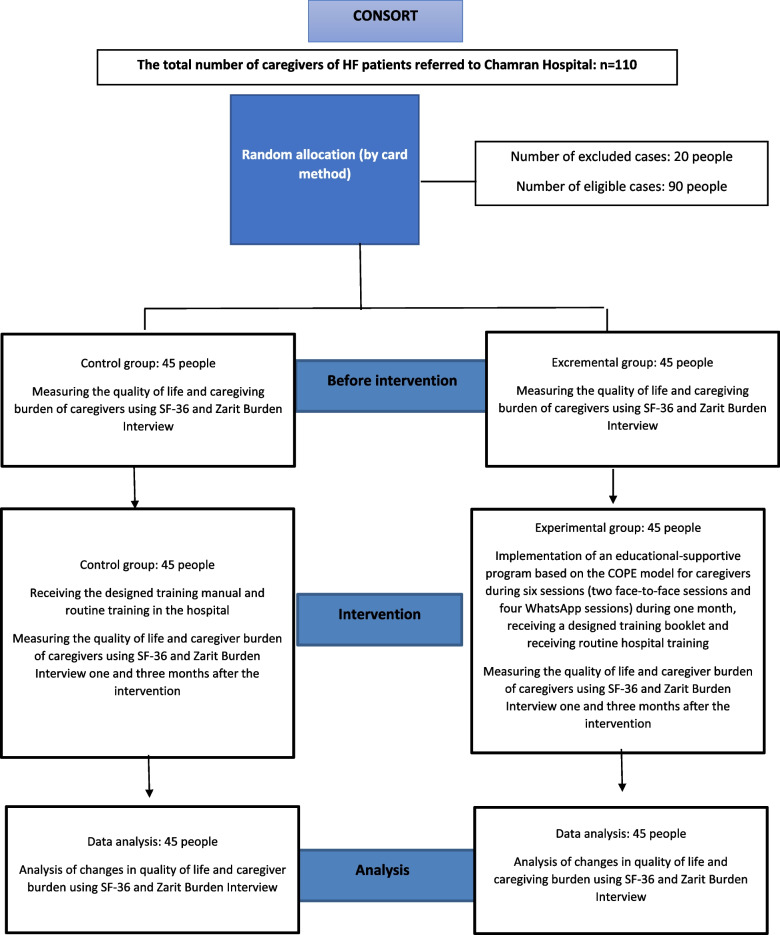
The research methodology

The present study aimed to determine the effectiveness of the supportive-educational program, based on the COPE care model, on the burden of care and quality of life of family caregivers of HF patients referred to Shahid Chamran Heart Educational, Medical and Research Center in Isfahan (Fig. 1). First, all the eligible caregivers referred to the clinic and wards of Chamran Hospital in Isfahan were selected. Also this study was presented to the caregivers, and if they wanted to participate, written informed consent was obtained from them.

It should be noted that the participants were assured that their non-participation in the study did not disrupt the treatment process of their patients, they could withdraw from the study at any time, and their personal information would remain confidential. Then the caregivers were randomly selected and placed in two experimental and control groups (*n* = 45 per group) using a card method [25, 26]. In this way, 45 cards with number one and 45 cards with number two were placed in a closed envelope, and on the day of the random distribution of research units, caregivers were asked to choose one card.

Then the people assigned number one were in the experimental group, and those assigned number two were in the control group. Next, by the card method, the people of the experimental group were divided into 9 blocks of 5 people, in the form of five cards number 1, five cards number 2, five cards number 3, five cards number 4, five cards Another number 5, five more cards number 6, five more cards number 7, five more cards number 8 and five more cards number 9 were placed in a closed envelope and on the day of random distribution, the people of the experimental group were asked to choose a card, the people who got number 1 in the first block, the people who got number 2 in the second block, the people who got number 3 in the third block, the people who got number 4 was awarded in the fourth block, the people who were awarded number 5 in the fifth block, the people who were awarded number 6 in the sixth block, the people who were awarded number 7 in the seventh block, the people who were awarded number 8 They were assigned in the eighth block and the people who were assigned number 9 were placed in the ninth block.

Because this study was single-blind study, only the researcher was aware of which test and control group each person was in, so none of the caregivers knew about this. Both groups (experimental and control) received routine training from the hospital about HF patients, including familiarization with the disease, its symptoms, drug treatment, dosage, side effects, symptoms of disease recurrence and visit time.. The experimental group received intervention in six sessions provided a training booklet and follow-up based on the COPE model forone month.

There were two face-to-face sessions in the hospital Within 60 min and four sessions on the WhatsApp platform Within 30 min. The follow-ups were also performed via WhatsApp, face-to-face, and telephone sessions. In these sessions, each caregiver's three main patient care-related problems, such as the patient's activity level,diet, ways to improve the quality of life and reduce the burden of care, and other issues were examined based on the COPE model.. The content of these sessions is listed in Table 1. At the end of the research, the training booklet was provided to the control group.

**Table 1:** The content of the sessions

Type and number of sessions	Content
Introduction session	1. Initial assessment 2. Getting to know the group members 3. Summarize the contents for the group members (introduction)
The first hospital session	1. Determining and prioritizing problems 2. Explaining the session plan 3. Explaining the problem-solving process based on the components of the COPE model 4. Teaching how to apply the problem-solving process based on the COPE model regarding the first problem, the model components include: Creativity: Since creativity is an intellectual and motivational activity, in this regard, the caregiver was encouraged to be able to see different aspects of the problem and come up with new ideas. Optimism: the caregiver was guided to have a positive and at the same time realistic and flexible attitude towards the problem-solving process Planning: To solve problems, the caregiver was directed to follow a regular and accurate approach to identify, solve problems. Expert advice: Caregivers seek to receive information from health professionals to understand different aspects of the disease, situations, problems, and care needs of their patients
The first WhatsApp call session	1. Checking the hours of reading the booklet according to the first problem. 2. Monitoring the program implementation 3. Answering the questions 4. Determining the second priority problem 5. Encourage the caregiver to read the materials related to the second problem before the second WhatsApp call session 6. Advice for determining the time of the next WhatsApp call
The second WhatsApp call session	1.Teaching how to apply the problem-solving process based on the COPE model regarding the second problem, the model components include: Creativity, Optimism, Planning, Expert advice 2. Creation of a brainstorm by the researcher for the caregivers 3. Explanation about the first determined problem and encouraging the caregiver to use the related materials in the booklet.
The second hospital session	1. A brief review of the problem-solving process based on the components of the COPE model 2. Reviewing the chapter on the second problem specified in the booklet 3. Completing the explanation about the second problem and encouraging the caregiver to use the related chapter in the booklet 4. Asking What they have done about the problem raised in the previous session 5. Providing more solutions regarding the second problem……
The third WhatsApp call session	1. A brief review of the problem-solving process based on the components of the COPE model 2. Teaching the problem-solving process about the third problem based on the COPE model 3. Completing the explanation about the second problem and encouraging the caregiver to use the related chapter in the booklet 4. Asking What they have done about the problem raised in the previous session. 5. Setting the time of the next WhatsApp call
The fourth WhatsApp session	1. Checking the hours of reading the booklet according to the first problem 2. Monitoring the program implementation, 3. Answering the questions, 4. Determining the time to complete the questionnaires

### Data collection tools

Demographic information checklist and SF-36 and Zarit caregiver burden were used in experimental and control groups before, immediately and three months after the intervention. The demographic information checklist included questions on age, gender, educational level, marital status, occupation, income level of the caregiver, client-caregiver relationship, and participation in caring for another person. SF-36 was designed in 1992 by Waroshrion in the United States [27]. This tool consists of 36 items that seek to evaluate the two-dimensional state of health in terms of physical and mental state, which is obtained by combining the scores of the eight health domains, including 1 physical functioning (PF), role physical (RP), bodily pain (BP), general health (GH), vitality (VT), social functioning (SF), role emotional (RE), and mental health (MH). The possible score range is 0 and 100. A score of zero indicates the lowest quality of life and vice versa, and a score below 50 indicates the low quality of life of the caregivers of these patients [27].

Zarit burden interview adapted from the questionnaire of a researcher named Zarit in 1980 [28] that consists of 22 items and assesses caregivers' individual, social, emotional, and financial dimensions [29]. Each item is scored based on a five-point Likert scale (Never (score 0), Rarely (score 1), Sometimes (score 2), Most of the time (score 3) and Always (score 4). Accordingly, the total score varies from 0 to 88. The sum of scores obtained by each caregiver indicates his/her care burden, The lower the score, the less the care burden. Scores 0, 1–30, 31–60, 61–88, and 88 indicate no, mild, moderate, severe, and the highest care burden, respectively [30]. This tool is adapted from the Zarit burden interview (1980). This questionnaire was designed to evaluate caregivers' psychological burden and has been used in many countries [28]. ZBI has been investigated by Navidian et al. (2008) and Shafizadeh Kholanjani et al. (2019). These two studies calculated the reliability of this questionnaire using the test–retest and Cronbach's alpha methods as 0.94, 0.96, and 0.91, 0.93, respectively [31, 32].

### Statistical analysis

Data analysis was carried out using SPSS software ver. 26. To analyze the demographic characteristics, chi-square (Fisher's exact) and independent-t tests were used, and independent-t, paired t-test, analysis of covariance (ANCOVA), repeated measures ANOVA and Kolmogorov–Smirnov test were used for variables of quality of life and burden of care. *P*-value < 0.05 was considered as the statistical significance level. Using the Chi-square test, it was determined that gender is a confounding variable, and after using covariance analysis, the effect of gender on the variables of quality of life and burden of care was not significant(*p* > 0.05).

## Results

### The demographic characteristics

There were 9(20) men and 36(80) women in the experimental group and 19(42.2) men and 26(57.8) women in the control group. There were 31(68.9) married people, 11(24.4) single people, 2(4.4) divorced people, and 1(2.2) widowed person in the experimental group, and 39(86.7) married people and 6(13.3) single people in the control group. A total of 12(26.7), 16()35.6), 5(11.1) individuals of the experimental group had middle school and below, diploma, an associate degree, bachelor's degree and above, and 18(33), 17(37.8), 4(8.9)and 6(13.3) individuals of the control group had middle school and below, diploma, associate degree, and bachelor's degree and above, respectively. There were 4(8.9) unemployed people, 19()42.2)( housewives, 18(40) employed people, 1(2.2) student and 3(7.6) retired people in the experimental group and 6(13.3) unemployed people, 17(37.8) housewives, 20(44.4) employed people and 2(4.4) retired people in the control group. The caregivers in the experimental group were parents, sisters, brothers, child, and spouse of the patients in 17(37.8), 1(2.2), 3(6.7), 17(37.8)(and 7(15.6)cases, and parents, brother, child, and spouse of the patients of the control group in 7(15.6), 2(4.4), 24(53.3)and 12(26.7) cases, respectively. Moreover, there were 30)66.7(people in the experimental group and 37(82.2)people in the control group. In addition to the participants, other caregivers also took care of their patients.

The mean age of the participants in the experimental and control groups was 41.56 ± 10.62 and 40.49 ± 10.80 years, respectively. The average one-month income of the caregivers in the experimental and control groups was 68.36 ± 104 and 77.27 ± 88.47 dollars, respectively. The average face-to-face patient care time in 24 h was 11.84 ± 5.14 and 10.44 ± 5.41 h in the experimental and control groups, respectively. there is no significant difference between the two groups in terms of the demographic information of the samples, except for gender. there is no significant difference between the two groups in terms of the demographic information of the samples(*p* > 0.05), except for gender(*p* < 0.05).

Table 2 shows the results of ANCOVA between the two experimental and control groups in terms of quality of life immediately and 3 months after the intervention after adjusting the pre-intervention values ​​and gender. Results showed that gender had no significant effect (*p* > 0.05).

**Table 2:** Analysis of covariance of the quality of life immediately and three months after the intervention between the two groups after adjusting the pre-intervention values ​ and gender

Variable	Analysis of covariance of the quality of life immediately after the intervention		Analysis of covariance of the quality of life three months after the intervention	
	**P**	**F**	**P**	**F**
**Group**	48.33	0.000	122.99	0.000
**Quality of Life**	85.47	0.000	86.15	0.000
**Gender**	0.021	0.885	0.52	0.470

Table 3 shows the results of ANCOVA between the two experimental and control groups in terms of caregiver burden immediately and 3 months after the intervention after adjusting the pre-intervention values ​​and gender. Results showed that gender had no significant effect (*p* > 0.05).

**Table 3:** Analysis of covariance of caregiver burden immediately and 3 months after the intervention between the two groups after adjusting the pre-intervention values ​​ and gender

Variable	Analysis of covariance of the caregiver burden immediately after the intervention		Analysis of covariance of the caregiver burden three months after the intervention	
	**P**	**F**	**P**	**F**
**Group**	88.24	0.000	217.87	0.000
**Caregiver burden**	171.39	0.000	137.07	0.000
**Gender**	1.16	0.283	2.15	0.146

Table 4 shows the frequency distribution of the research subjects in terms of the quality of life and its dimensions before, immediately and three months after the intervention.

**Table 4:** Frequency distribution of research subjects in terms of the levels of quality of life of the two groups at three time periods

**Quality of life and its dimensions**	**Level**	**Time**	**Control**	**Experimental**
			**Number (Percentage)**	**Number (Percentage)**
**Quality of Life**	**Low**	**Before**	14 (31.1)	7 (15.6)
	**High**		31 (68.9)	38 (84.4)
	**Low**	**Immediately after**	17 (37.8)	3 (6.7)
	**High**		28 (62.2)	42 (93.3)
	**Low**	**Three months later**	19 (42.2)	0 (0)
	**High**		26 (57.8)	45 (100)
**Physical functioning**	**Low**	**Before**	15 (33.3)	8 (17.8)
	**High**		30 (66.7)	37 (82.2)
	**Low**	**Immediately after**	16 (35.6)	5 (11.1)
	**High**		29 (64.4)	40 (88.9)
	**Low**	**Three months later**	19 (42.2)	5 (11.1)
	**High**		26 (57.8)	40 (88.9)
**Role physical**	**Low**	**Before**	31 (68.9)	32 (71.1)
	**High**		14 (31.1)	13 (28.9)
	**Low**	**Immediately after**	32 (71.1)	15 (33.3)
	**High**		13 (28.9)	30 (66.7)
	**Low**	**Three months later**	31 (68.9)	12 (26.7)
	**High**		14 (31.1)	33 (73.3)
**Role emotional**	**Low**	**Before**	29 (64.4)	25 (55.6)
	**High**		16 (35.6)	20 (44.4)
	**Low**	**Immediately after**	29 (64.4)	9 (20)
	**High**		16 (35.6)	36 (80)
	**Low**	**Three months later**	28 (62.2)	9 (20)
	**High**		17 (37.8)	36 (80)
**Fatigue or vitality**	**Low**	**Before**	20 (44.4)	10 (22.2)
	**High**		20 (55.6)	35 (77.8)
	**Low**	**Immediately after**	20 (44.4)	5 (11.1)
	**High**		25 (55.6)	40 (88.9)
	**Low**	**Three months later**	16 (35.6)	1 (2.2)
	**High**		29 (64.4)	44 (97.8)
**Mental health**	**Low**	**Before**	14 (31.1)	8 (17.8)
	**High**		31 (68.9)	37 (82.2)
	**Low**	**Immediately after**	12 (26.7)	5 (11.1)
	**High**		33 (73.3)	40 (88.9)
	**Low**	**Three months later**	12 (26.7)	0 (0)
	**High**		33 (73.3)	45 (100)
**Social functioning**	**Low**	**Before**	17 (37.8)	17 (37.8)
	**High**		28 (62.2)	28 (62.2)
	**Low**	**Immediately after**	20 (44.4)	8 (17.8)
	**High**		25 (55.6)	37 (82.2)
	**Low**	**Three months later**	28 (62.2)	3 (6.7)
	**High**		17 (37.8)	42 (93.3)
**Bodily pain**	**Low**	**Before**	10 (22.2)	9 (20)
	**High**		35 (77.8)	36 (80)
	**Low**	**Immediately after**	13 (28.9)	2 (4.4)
	**High**		32 (71.1)	43 (95.6)
	**Low**	**Three months later**	14 (31.1)	0 (0)
	**High**		31 ((68.9)	45 (100)
**General health**	**Low**	**Before**	45 (100)	0 (0)
	**High**		0 (0)	45 (100)
	**Low**	**Immediately after**	14 (31.1)	3 (6.7)
	**High**		31 (68.9)	42 (93.3)
	**Low**	**Three months later**	14 (31.1)	0 (0)
	**High**		31 (68.9)	45 (100)

**Fig. 2:**
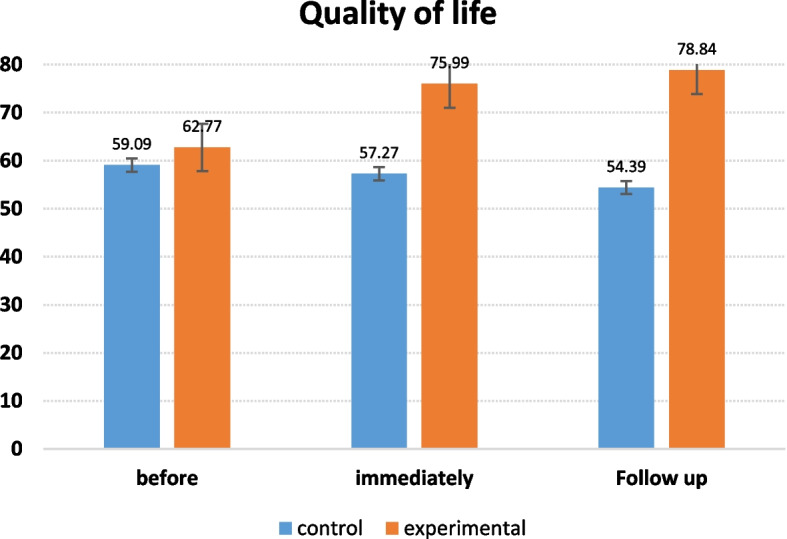
The rectangular figure of the mean quality of life in the control and experimental groups at different times of the intervention

**Fig. 3:**
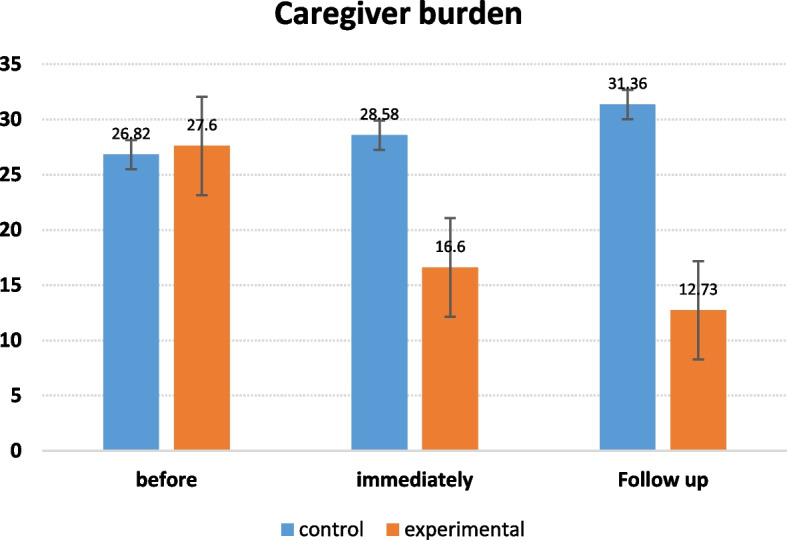
The rectangular figure of the mean burden of care in the control and experimental groups at different times of the intervention

Investigating the effect of the COPE intervention on quality of life and burden of care three times: Rectangular Figs. 2 and 3 show the changes in quality of life and caregiver burden during three time periods, including before, immediately and three months after the intervention.

## Discussion

The present study aimed to determine the effectiveness of the supportive-educational program, based the on COPE care model, on the quality of life and caregiver burden of family caregivers of HF patients.

The present research showed that the four-component COPE model for caregivers of HF patients is suitable for each caregiver's problem during care. The COPE model is distinguished from other approaches by its focus on caregivers' problems as the main and important key in care and treatment [21].

The studied components include quality of life and burden of care, which were measured separately. After assessing the amount of caregivers' information regarding these two components, the researcher gave the necessary training based on the COPE model. It can be concluded (Fig. 2) that the supportive-educational intervention, based on the COPE model, improved the quality of life in the experimental group compared to the control group. In this regard, Banihashemi et al. (2020) showed that interventions of cognitive behavioral therapy, reality therapy, and acceptance and commitment therapy increase the quality of life of caregivers of patients with chronic diseases [10].

Fagerström et al. [33] performed a cross-sectional cohort study on 3444 samples to analyze the conditions of elderly family caregivers with a focus on health-related quality of life and pain in 2020. This study was conducted in Sweden from 2001 to 2004, and the results showed that the most influencing factor on the quality of life of family caregivers of older people was their pain and mental health, respectively [34]. Akbari et al. [34] and Abiolahazan et al. [35] showed that the care of patients with chronic diseases has negative effects on their families' quality of life and the quality of their patient care [35, 36]. The study of Sajadi et al. [36] found that implementing supportive educational interventions, including therapeutic hope, increased the quality of life of caregivers of hemodialysis patients [37]. The study of Fallahzadeh & Balanian [37] investigated the SF-36 dimensions in the quality of life of postmenopausal women referring to health centers in Yazd. The SF-36 was used in this study, and the highest and lowest mean quality of life score of menopausal women was related to the ​​pain, and of ​​role physical dimension, respectively [38]. The study of Khodaveisi et al.  [38] the health belief model is useful for improving people's understanding [39]. These studies were consistent with the present study.

The inconsistent studies were mentioned below: Mahmoudi et al. [39] carried out a study to investigate the effect of Hatha yoga exercises on the quality of life of patients undergoing hemodialysis in 2018. The data collection instrument in this study included SF-36. The results showed that hatha yoga caused significant changes in the fatigue or vitality domain of patients' quality of life undergoing hemodialysis [40]. Also, Soleimani et al. [40] investigated the effect of type D personality traits on addiction severity and quality of life in people undergoing methadone maintenance treatment. They found a significant relationship between studied variables with vitality, fatigue, and general health, and no significant relation with bodily pain [33]. This discrepancy between these studies and the current study is attributed to the difference in sample size, type of patients, type of statistical analysis, type of study, and intervention, which is recommended to conduct further studies.

The mean caregiver burden score in the experimental and control groups was equal before the intervention and there was no significant difference. The mean quality of life score in the experimental group was significantly higher than the control group one month after the intervention (*p* = 0.000). Moreover, the quality of life score in the experimental group was significantly higher than the control group three months after the intervention. It can be inferred from the results (Fig. 3) that the supportive-educational intervention, based on the COPE model, improved the caregiver burden component in the experimental group compared to the control group. Most people in the experimental and control groups had mild caregiver burden (0–30) before, immediately, and three months after the intervention. In a study on the effect of group training on the caregiver burden of caregivers of cancer children, Ali Akbarian et al. (2019) reported that group training empowers parents to take care of their children, reducing the caregiver burden of these caregivers [14].

In a study of the effect of spiritual self-care training on the caregiver burden of mothers of children undergoing heart surgery in a pediatric cardiac intensive care unit, Delir et al. (2019) showed that spiritual self-care training reduces the caregiver burden in mothers of children undergoing heart surgery [41], Both studies are consistent with the present study. In a study of the perceived stress and caregiver burden in elderly caregivers: the moderating role of resilience, Jafari et al. (2022) showed that elderly caregivers experience a moderate to high caregiver burden [42]. In a study of the effect of web-based health information on the caregiver burden of family caregivers of dementia patients, Salehinejad et al. (2018) showed these caregivers experience a moderate burden of care [43], which is not consistent with the present study. This inconsistency may be due to the difference in the study population of patients, type of interventions, and study. It is thus recommended to conduct further relevant studies.

Results of various studies on the caregivers of patients with chronic diseases show a close relationship between the quality of life and caregiver burden. In this regard, Jafari et al. (2018) showed that caregivers of hemodialysis patients endure a high care burden, and this pressure has a negative effect on their quality of life [44]. Abbasi et al. (2020) showed that the caregivers of cancer patients are susceptible to caregiver burden that affects their quality of life [45]. The results of Doris et al.'s study (2020) showed a reduction in the quality of life and the high caregiver burden of caregivers of patients needingof palliative care [46]. All these studies are consistent with the present study. Therefore, it is recommended in all studies to identify the needs and problems of caregivers of patients and to provide appropriate solutions and support to caregivers to reduce caregiver burden and thus increase the quality of life of these caregivers and subsequently their patients [47].

### Limitations

In this study, there were limitations such as receiving information from informal spaces by the caregivers of the patients and creating a disruption in the process of teaching them in the training sessions and the limited sample group to a province. Therefore, generalizing the study results should be done with caution due to the difference in culture regarding the understanding of concepts such as quality of life and caregiver burden.

### Suggestions

Since the educational-supportive program based on the COPE model in the present study had a significant effect on the existing content and structure, researchers can conduct further studies on the quality of life and caregiving burden of this group of caregivers. One of the important duties of nurses is to maintain and improve the health level of patients and their caregivers, so it is necessary to implement educational-supportive programs in hospitals, after the discharge of patients, These programs are family-oriented remotely for their caregivers. Continue using social messengers in voice or text. Also, according to the results of this research, the COPE model can be used to improve different aspects of the life of patient caregivers, such as quality of life and care burden. It can be said that educational-support programs improve the mental, social, and physical level of caregivers, and as a result, better care at home, reducing treatment costs, reducing hospitalization, and increasing patient survival. Generally, reducing the negative effects on caregivers translates into their patients.

## Conclusion

The supportive-educational program, based on the COPE model, is a caregiver-centered program that increases caregivers' knowledge and awareness by using the four components of creativity, optimism, planning, and expert advice, which subsequently positively affects various physical and psychological aspects of caregivers.

## Data Availability

The datasets generated and analyzed during the current study are not publicly available due to the identity information contained in the data but they can be obtained from the corresponding author on reasonable request by deleting this information.

## Abbreviations

COPE: Creativity Optimism Planning Expert Information
HF: Heart failure
SF-36: The Short from Health Survey
RCT: Randomized controlled Trial
